# Editorial: Machine learning-based adaptive radiotherapy treatments: From bench top to bedside

**DOI:** 10.3389/fonc.2023.1188788

**Published:** 2023-04-19

**Authors:** Jiahan Zhang, Yang Sheng, Justin Roper, Xiaofeng Yang

**Affiliations:** ^1^ Department of Radiation Oncology, Emory University, Atlanta, GA, United States; ^2^ Department of Radiation Oncology, Duke University, Durham, NC, United States

**Keywords:** adaptive radiotherapy, machine learning, image segementation, treatment planning, image registration

## Introduction

Radiation therapy aims to control malignant and less commonly benign diseases while preserving the surrounding healthy tissues. Standard courses of radiation therapy last up to six weeks, during which time anatomical changes are often anticipated due to tumor shrinkage and the day-to-day variations of organ filling and patient positioning. Historically, clinicians have compensated for these variations by adding generous margins around target volumes to prevent a geometric miss but at the expense of increased radiation dose to the healthy tissues. One alternative is adaptive radiotherapy where the patient receives customized treatment based on the “anatomy of the day.” This approach reduces the need for large margins by directly accounting for the inter-fraction variations and consequently better spares the healthy tissues. Adaptive radiotherapy has been an active research area for some time and finally has been commercialized and implemented in some radiotherapy clinics, due in large part to machine learning. In this Research Topic “Machine Learning-Based Adaptive Radiotherapy Treatments: From Bench Top to Bedside”, machine learning applications in various stages of the adaptive radiotherapy workflow are covered, including image registration, segmentation, treatment planning, and clinical decision support.

## Topics covered in this research topic

AI-driven image segmentation: Naser et al., Domoguen et al., Xia et al.

Treatment-time image processing/correction: Yang et al., Cao et al.

Automated treatment planning: Fredén et al., Pogue et al.

Clinical decision support *via* dosiomics and radiomics: Kraus et al.

Online adaptive planning workflow validation: Chen et al., Magallon-Baro et al.

## Papers included in this research topic

Naser et al. used auto-segmentation to help define the skeletal muscle index (SMI) and calculate sarcopenia, a prognostic factor for head-and-neck cancer (HNC) patients. The auto-segmentation approach substantially improved the efficiency in determining sarcopenia and proved effective in predicting patient survival. The proposed model could potentially assist in clinical decision-making for HNC treatments.

Domoguen et al. designed a deep learning architecture for the task of auto-segmenting nasopharyngeal carcinoma (NPC) target volumes. This model is based on UNet-2.5D and has been enhanced with multi-scale training and semi-supervised pre-training to improve training efficiency. With a small training/validation dataset, the proposed method demonstrated improved performance at NPC target volume segmentations as compared to the current state-of-the-art methods, indicating efficient use of limited data.

Xia et al. developed an attention-based UNet model to auto-segment parotid neoplasms, a relatively rare form of HNC. The authors reported an average Dice similarity coefficient (DSC) of 0.88 for both parotids. The performance of the proposed model was comparable to human observers (3 radiologists).

Yang et al. compared two approaches for enhancing the image quality of cone-beam CT (CBCT) images: 1) deformable registration of planning CT images to CBCT images, and 2) synthetic CT images derived from CBCT images. The authors found that the auto-segmented contours based on synthetic CT images achieved significantly higher DSCs for bladder, rectum, spinal cord, and femoral heads, compared with contours segmented on deformed planning CT images. This study validated the efficacy of synthetic CT images for auto-segmenting pelvic anatomy.

Cao et al. proposed a novel method to eliminate metal artifacts by synthesizing CT from mega-voltage CBCT (MVCBCT). They implemented a cycle-consistent generative adversarial network (CycleGAN) to synthesize metal artifact-free CT images. The process successfully eliminated metal artifacts. Further, Gamma analysis of the dose matrices calculated based on planning CT and synthesized CT confirmed the dose calculation accuracy.

Fredén et al. studied the effects of adaptive radiation therapy on dose painting treatments. The authors compared the tumor control probability (TCP) of the adaptive workflow and the conventional workflow and found that adaptive workflow consistently achieved target coverage, albeit with marginal improvements in the TCP.

Pogue et al. investigated an automated adaptive planning workflow for accelerated partial breast irradiation with an emphasis on cardiac sparing. A commercial adaptive radiotherapy platform, Varian Ethos, was used to test the workflow. Two physicians evaluated the auto-generated plans and found at least 95% of cases were clinically acceptable. Additionally, the auto-generated plans improved cardiac sparing compared with the previous manual plans.

Kraus et al. developed a model to predict radiation-induced pneumonitis using both dosiomic features and radiomic features. With both sets of features, the model achieved an area under the ROC curve (AUC) of 0.79, suggesting that the model could effectively predict pneumonitis before treatment and help guide clinical decision-making for at-risk patients.

Chen et al. studied the feasibility of using auto-segmented contours directly for cervical cancer VMAT planning. They evaluated plan metrics for plans created based on auto segmentations (AS-VMAT) and compared that with manual segmentations (MS-VMAT) results. While for most organs at risk (OARs), the difference between AS-VMAT and MS-VMAT was not significant, MS-VMAT plans achieved better rectum sparing. The study concluded that auto-segmented contours, especially for organs in close proximity to the target volume, need to be examined carefully to ensure plan quality.

Magallon-Baro et al. explored the feasibility of adaptive treatment planning for pancreas stereotactic body radiotherapy (SBRT) with contours deformed from planning CTs onto treatment CBCTs. Two commercial deformable registration methods were tested. Replanning with unedited, deformed contours resulted in slightly worse results due to inaccuracies in contours near the target volumes. However, the automated plans still outperformed the non-adapted plans.

## Conclusions and outlook

Adaptive therapy is in a crucial phase, transitioning from the bench top to the bedside. With the first generation of commercial adaptive treatment machines and solutions already in some radiation oncology centers, there is emerging expertise in the clinical implementation of adaptive planning workflows. This invaluable clinical knowledge from incorporating adaptive therapy into routine clinical practice will undoubtedly encourage related research activities to enhance accuracy and efficiency, which further promotes the clinical implementation of adaptive therapy. In parallel, researchers are making strides in developing advanced adaptive treatment technologies based on information from various imaging modalities. This Article Collection showcases both the practical aspects of clinical applications of AI-driven modern adaptive therapy workflows and cutting-edge technological advancements in this domain. The adaptive radiotherapy treatments that clinicians have long dreamed of are now gradually becoming a clinical reality.

## Author contributions

All authors listed have made a substantial, direct, and intellectual contribution to the work and approved it for publication.

